# Repeated intravenous thrombolysis in recurrent ischemic stroke within 3 months: a systematic review

**DOI:** 10.1186/s12883-023-03472-4

**Published:** 2023-11-27

**Authors:** Si-Yuan Wen, Fei-Fei Chen, Xiang-Ting Chen, Qian Zhang, Chang-Qing Zhou

**Affiliations:** Department of Neurology, Bishan Hospital of Chongqing Medical University, Chongqing, China

**Keywords:** Recurrent ischemic Stroke, Repeated intravenous thrombolysis, Within 3 months, Symptomatic intracranial Hemorrhage

## Abstract

**Background:**

Repeated intravenous thrombolysis (RIVT) within 3 months is an off-guideline therapy, however, may be an effective and safe way to treat early recurrent ischemic stroke. This study was conducted to assess the potential influencing factors on the efficacy and safety of RIVT in recurrent ischemic stroke within 3 months and to explore the strategy of RIVT within 3 months.

**Methods:**

PubMed, Cochrane Library, Embase, China National Knowledge Infrastructure, and Wanfang Database were searched for cases of RIVT in recurrent ischemic stroke within 3 months up to February 1, 2023. Clinical characteristics were compared and analyzed between the good-outcome and poor-outcome groups and between the symptomatic intracranial hemorrhage (sICH) and non-sICH groups respectively.

**Results:**

A total of 16 studies including 24 cases of RIVT within 3 months were retrospectively analyzed in the present study. The patients’ ages ranged from 42 to 87 years (median 73.5 years) and the intervals between thrombolysis were from 0.25 to 90 days (median 9.5 days). Comparing the clinical characteristics between the good-outcome group and the poor-outcome group, no statistically significant differences were found (*P* > 0.05), but the differences in baseline National Institutes of Health stroke scale (NIHSS) score of the recurrent stroke (*P* = 0.056) and good outcome after the previous IVT (*P* = 0.054) nearly reached statistical significance. Comparing the data between the non-sICH group and the sICH group, statistically significant differences were found in terms of the proportion of cardiogenic embolism (*P* = 0.036), baseline NIHSS score in the recurrent stroke (*P* = 0.007) and the interval between thrombolysis (*P* = 0.041), but no significant difference was found by regression analysis.

**Conclusion:**

In patients with recurrent ischemic stroke within 3 months, those with a good outcome after the previous IVT and a low baseline NIHSS score in the recurrent stroke may be considered for RIVT, whereas those with a high baseline NIHSS score, a short interval between thrombolysis, and cardiogenic embolism may suffer a higher risk of sICH. Due to sample size and publication bias, more studies with larger sample sizes and more rigorous designs are needed to confirm this conclusion.

**Supplementary Information:**

The online version contains supplementary material available at 10.1186/s12883-023-03472-4.

## Background

Acute ischemic stroke (AIS) is the most common type of stroke. The key to the treatment of AIS is to recanalize the blocked cerebral blood vessels with the least possible delay and improve the symptoms of neurological deficits. At present, the main treatment methods are still intravenous thrombolysis (IVT) and mechanical thrombectomy. However, due to the limitations of medical conditions, not all medical institutions can implement mechanical thrombectomy, so IVT is still the most important treatment for AIS [1–3].

For a long time, a history of AIS within 3 months is regarded as a contraindication for thrombolysis therapy in the AIS treatment guidelines in many countries. The European Stroke Organization (ESO) indicated in their guidelines that there is insufficient evidence to recommend intravenous thrombolysis in patients with a history of AIS within the past 3 months [1]. American Heart Association (AHA) / American Stroke Association (ASA) considered IVT may be harmful in patients with AIS within the last 3 months [2]. Chinese guidelines also pointed out that a history of stroke in the past 3 months is a contraindication for intravenous thrombolysis [3]. The reason is that IVT may increase the risk of intracranial hemorrhage in patients with a history of stroke within the past 3 months [4]. Although the recurrence rate of AIS has gradually decreased through the continuous standardization of prevention and treatment in recent years, there are still many patients with AIS recurrence within 3 months in clinical practice. If all these patients are regarded as having contraindications for IVT, they will miss the opportunity of this effective vascular recanalization treatment. In recent years, some studies have shown that re-administration of intravenous thrombolysis may be effective and safe in some selected patients with recurrent AIS within 3 months [5–7]. But the factors that influence the effectiveness of repeated intravenous thrombolysis (RIVT) remain unclear. This study reviewed the cases of RIVT within 3 months published worldwide to analyze the characteristics of successful cases of RIVT and to explore the strategy of RIVT within 3 months.

## Methods

The Preferred Reporting Items for Systematic Reviews and Meta-Analyses (PRISMA) guideline was used for developing this review [8].

### Search strategy

Two independent reviewers (Wen SY and Chen FF) performed the search. PubMed, Cochrane Library, Embase, China National Knowledge Infrastructure, and Wanfang Database were searched up to February 1, 2023. Types of articles included but were not limited to case reports, case series, randomized controlled trials, cohort studies, etc. The search strategy was developed according to “recurrent ischemic stroke”, “intravenous thrombolysis” and “within 3 months” and their related text vocabulary and MeSH terms ((“stroke” or “cerebrovascular accident”) and (“thrombolysis” or “IVT” or “alteplase” or “tenecteplase” or “urokinase”) and (“three months” or “recurrent” or “repeated”)). Studies of RIVT within 3 months of recurrent AIS were screened from the search results, and repeated irrelevant articles were eliminated.

### Eligibility criteria

All potentially relevant studies were screened for eligibility by two independent reviewers (Chen XT and Zhang Q). These studies met the following inclusion criteria: (1) Recurrent AIS patients were confirmed and diagnosed. (2) IVT was performed both in the index stroke and the recurrent stroke, and the interval between two IVT was no more than 90 days. (3) Containing data for clinical manifestation, treatment, and outcome of the patient was addressed. Studies without individual-level data, review articles, commentary, and other irrelevant articles were excluded. The quality of included studies was evaluated using the Joanna Briggs Institute (JBI) Critical Appraisal Checklist for Analytical Cross Sectional Studies. And any disagreements were discussed and resolved by a third investigator (Zhou CQ).

### Data collection

Two independent reviewers (Wen SY and Chen FF) conducted data collection. Names of the first author, the year of publication, gender and age of patients, involved vessels, TOAST classification, baseline National Institutes of Health stroke scale (NIHSS) score, onset-to-treatment (OTT) time, the interval between thrombolysis, drug administration, modified Rankin scale (mRS) at 90 days were extracted from all included studies. All information was filled out on a pre-structured form.

### Index for assessment

The NIHSS score was used to assess the severity of symptoms, and the mRS score was used to evaluate the prognosis of patients. The main efficacy index is whether the patient finally obtained a good clinical outcome after RIVT, and the main safety index is whether symptomatic intracranial hemorrhage (sICH) occurred and mortality. We defined a good clinical outcome as the mRS score at 90 days ≤ 2 points after IVT, if no mRS was reported in the case, as a decrease in the NIHSS at discharge ≥ 4 points from the baseline NIHSS score.

### Statistical methods

Statistical analysis was performed using IBM SPSS Statistics 27.0 software. In the efficacy assessment, cases were divided into a good-outcome group and a poor-outcome group based on the clinical outcome after RIVT for comparative analysis. In the safety assessment, cases were divided into an sICH group and a non-sICH group based on the occurrence of sICH after RIVT for comparative analysis. The amount of measurement data is small and does not fit normality and homogeneity of variance, so those data were shown as [the median (minimum value, maximum value)], and the Mann-Whitney U test is used for comparison between groups. Enumeration data were shown as [the number of cases (percentage)], and the Fisher’s precision probability test was used for comparison between groups. Logistic regression analysis was used to analyze the possible influencing factors. The difference was considered statistically significant when *P* < 0.05.

## Results

### Included studies

**Fig. 1:**
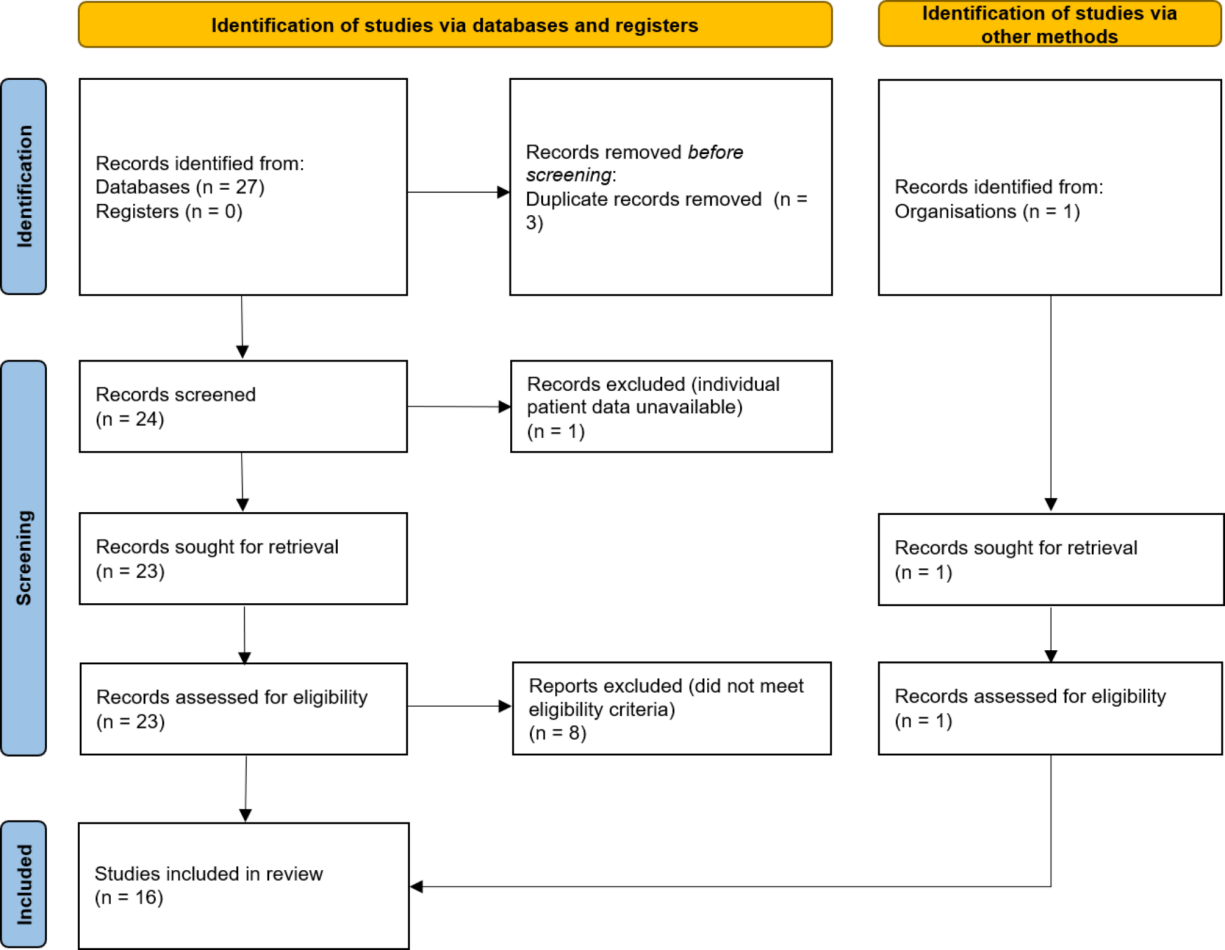
PRISMA flow diagram illustrating the process of study selection

After screening the search results, 15 studies were included (The flow diagram of study selection is shown in Fig. 1). In addition, we included a case report of RIVT within 3 months in our hospital [9]. The clinical information of each case is shown in Table 1 [9–24]. Since case 9(10) was performed 4 times of IVT (The interval between the 1st and 2nd thrombolysis is more than 3 months. The intervals between the 2nd and 3rd and the 3rd and 4th thrombolysis are less than 3 months. Thus, the data of the 1st thrombolysis was excluded in the statistical analysis) and case 20(21) was performed 3 times of IVT (The intervals between the 1st and 2nd and the 2nd and 3rd thrombolysis are less than 3 months), those cases were regarded as two cases respectively for subsequent statistical analysis. Eventually, a total of 24 cases of RIVT in recurrent AIS within 3 months were included in this study.

### Population characteristics in the included cases

Among the included cases, there were 14 males (58.33%) and 10 females (41.67%), aged 42–87 years, with a median age of 73.5 years, and the median interval between thrombolysis was 9.5 days. In terms of TOAST classification, there were 9 cases (37.50%) of large artery atherosclerotic (LAA), 8 cases (33.33%) of cardiogenic embolism (CE), 5 cases (20.83%) of small artery occlusion (SAO), and 2 cases (8.33%) in which TOAST classification was not reported. The TOAST classification was the same for both the index stroke and the recurrent stroke. In the index stroke, anterior cerebral circulation was involved in 16 cases (66.67%), posterior cerebral circulation was involved in 6 cases (25.00%), and 2 cases (8.33%) did not report the involved artery. In the recurrent stroke, anterior cerebral circulation was involved in 19 cases (79.17%), posterior cerebral circulation was involved in 4 cases (16.67%), and 1 case (4.17%) did not report the involved artery. The baseline NIHSS score of the index stroke was 2–19 points (5 cases did not report) and the baseline NIHSS score of the recurrent stroke was 2–24 points (2 cases did not report). Except for case 23 which used tenecteplase during the RIVT, all other cases used alteplase as the thrombolytic drug. In addition, mechanical thrombectomy was performed together with IVT in case 12 for the recurrent stroke.

After the previous IVT, 22 cases (91.70%) obtained good clinical outcomes and 2 cases (8.30%) obtained poor clinical outcomes. After the RIVT, 18 cases (75.00%) obtained good clinical outcomes and 6 cases (25.00%) obtained poor clinical outcomes, of which 2 cases had poor clinical outcomes after both two IVT. There was no case of sICH after the previous IVT. Three cases (12.50%) occurred sICH after the RIVT and case 13 died of pulmonary infection during hospitalization.

**Table 1:** Characteristics of included cases

Study	No.	Sex	Age	TOAST	Index Stroke						Interval (day)	Recurrent Stroke					
					Artery	NIHSS	OTT (min)	Drug	sICH	Good outcome		Artery	NIHSS	OTT (min)	Drug	sICH	Good outcome
Chen et al. [9] 2023	1	F	80	CE	L VA + BA	10	50	alteplase 0.9 mg/kg	NO	YES	9	L VA + BA + PCA	12	140	alteplase 0.6 mg/kg	NO	YES
Černík et al. [10] 2021	2	M	74	LAA	BA	6	35	alteplase 0.9 mg/kg	NO	YES	2	BA + R VA	24	44	alteplase 0.9 mg/kg	NO	NO
	3	M	52	SAO	BA	5	44	alteplase 0.9 mg/kg	NO	YES	11	BA	8	33	alteplase 0.9 mg/kg	NO	YES
Bouchal et al. [11] 2021	4	M	58	LAA	R MCA	13	90	alteplase 0.9 mg/kg	NO	YES*	10	R MCA	14	60	alteplase 0.9 mg/kg	NO	YES
Xiao et al. [12] 2017	5	M	52	LAA	L MCA	14	90	alteplase 0.9 mg/kg	NO	YES*	9	L MCA	12	50	alteplase 0.9 mg/kg	NO	YES*
Laible et al. [13] 2015	6	M	80	CE	BA	19	NR	alteplase 0.9 mg/kg	NO	YES	30	L MCA	18	> 420	alteplase 0.9 mg/kg	NO	NO
	7	F	51	SAO	L MCA	11	350	alteplase 0.9 mg/kg	NO	NO	73	L MCA	14	119	alteplase 0.9 mg/kg	NO	NO
	8	M	48	CE	L MCA	2	NR	alteplase 0.9 mg/kg	NO	YES	66	L MCA	2	220	alteplase 0.9 mg/kg	NO	YES
Mechtouff et al. [14] 2015	9	M	42	LAA	L MCA	6	226	alteplase	NO	YES	14(2nd -3rd)	L MCA	2	150	alteplase	NO	YES
	10	M	42	LAA	L MCA	2	150	alteplase	NO	YES	7(3rd -4th)	L MCA	7	110	alteplase	NO	YES
Qureshi et al. [15] 2015	11	M	73	LAA	NR	NR	NR	alteplase 0.9 mg/kg	NO	YES*	6	R ICA	6	NR	alteplase 0.9 mg/kg	NO	YES
	12	M	54	NR	L MCA	NR	NR	alteplase 0.9 mg/kg	NO	YES*	49	L MCA	2	NR	alteplase 0.9 mg/kg	NO	YES
Sposato et al. [16] 2013	13	F	76	CE	R MCA	18	120	alteplase 0.9 mg/kg	NO	YES*	4	L MCA	22	240	alteplase 0.9 mg/kg	YES	NO
Yoo et al. [17] 2013	14	F	73	CE	L MCA	12	70	alteplase 0.9 mg/kg	NO	YES	90	L MCA	12	55	alteplase 0.9 mg/kg	NO	YES
Alhazzaa et al. [18] 2013	15	F	81	CE	R MCA	NR	NR	alteplase 0.9 mg/kg	NO	NO*	6	R MCA	23	NR	alteplase 0.9 mg/kg	YES	NO
	16	M	83	LAA	R MCA	NR	NR	alteplase 0.9 mg/kg	NO	YES*	70	R MCA	9	NR	alteplase 0.9 mg/kg	NO	NO
	17	M	87	LAA	R MCA	NR	NR	alteplase 0.9 mg/kg	NO	YES*	70	R MCA	4	NR	alteplase 0.9 mg/kg	NO	YES
Brigo et al. [19] 2012	18	M	73	SAO	NR	4	240	alteplase 0.9 mg/kg	NO	YES*	2	NR	12	60	alteplase 0.9 mg/kg	NO	YES*
Cappellari et al. [20] 2012	19	F	75	CE	R MCA	4	175	alteplase 0.9 mg/kg	NO	YES*	2	L MCA	15	35	alteplase 0.9 mg/kg	NO	YES*
Nicoli et al. [21] 2011	20	F	77	SVO	R MCA	12	100	alteplase	NO	YES*	7	R MCA	7	95	alteplase	NO	YES*
	21	F	77	SVO	R MCA	7	95	alteplase	NO	YES*	10(2nd -3rd)	R MCA	10	105	alteplase	NO	YES*
Sauer et al. [22] 2010	22	F	84	NR	PCA	9	120	alteplase	NO	YES	90	MCA	12	120	alteplase	NO	YES
Smadja et al. [23] 2009	23	M	74	CE	BA	6	240	alteplase 0.9 mg/kg	NO	YES*	0.25	BA	22	60	tenecteplase 0.4 mg/kg	YES	YES*
Topakian et al. [24] 2005	24	M	50	LAA	R MCA	9	120	alteplase 90 mg	NO	YES*	4	R MCA	10	85	alteplase 50 mg	NO	YES

Abbreviations: OTT = onset-to-treatment time; sICH = symptomatic intracranial hemorrhage; NIHSS = National Institute of Health stroke scale; NR = not reported; LAA = large artery atherosclerotic; CE = cardiogenic embolism; SAO = small artery occlusion; L = left; R = right; MCA = middle cerebral artery; PCA = posterior cerebral artery; VA = vertebral artery; BA = basilar artery.

Good clinical outcome: * indicates a decrease in the NIHSS score at discharge ≥ 4 points from the baseline, others indicate the mRS score at 90 days ≤ 2 points after IVT.

### Assessment of efficacy

Comparing the differences between the good-outcome group and the poor-outcome group under different influencing factors (Table 2), the results showed no statistical significance between the two groups (*P* > 0.05). The proportion of good outcomes after the previous IVT (*P* = 0.054) and baseline NIHSS score of the recurrent stroke (*P* = 0.056) showed borderline significance between the two groups, which suggests that these two factors may have an effect on clinical outcomes after RIVT.

**Table 2:** Comparison between cases with good and poor clinical outcomes of RIVT

Influencing factors	Good-outcome group (18 cases)	Poor-outcome group (6 cases)	*P* value
Male [cases (%)]	11 (61.11%)	3 (50.00%)	0.494
Age (year) [median (min, max)]	69.25 (42, 87)	78.00 (51, 83)	0.156
ACC involved in the index stroke [cases (%)]	12 (75.00%)	4 (66.67%)	0.541
ACC involved in the recurrent stroke [cases (%)]	14 (82.35%)	5 (83.33%)	0.73
TOAST classification			
LAA [cases (%)]	7 (43.75%)	2 (33.33%)	0.523
CE [cases (%)]	5 (31.25%)	3 (50.00%)	0.369
SAO [cases (%)]	4 (25.00%)	1 (16.67%)	0.581
Baseline NIHSS of the index stroke [median (min, max)]	7.00 (2, 14)	14.50 (6, 19)	0.1
Baseline NIHSS of the recurrent stroke [median (min, max)]	10.40 (2, 22)	18.00 (8, 24)	0.056
OTT of the previous IVT (min) [median (min, max)]	106.67 (44, 240)	120.00 (35, 350)	0.953
OTT of the RIVT (min) [median (min, max)]	85.00 (33, 220)	179.50 (44, 420)	0.221
The interval between two IVT (day) [median (min, max)]	9.50 (0.25, 90)	18.00 (2, 73)	0.974
Good outcome after previous IVT [cases (%)]	18 (100.00%)	4 (66.67%)	0.054

Abbreviations: ACC = anterior cerebral circulation; LAA = large artery atherosclerotic; cardiogenic embolism; SAO = small artery occlusion; OTT = onset-to-treatment time; IVT = intravenous thrombolysis.

### Assessment of safety

Comparing the differences between the non-sICH group and the sICH group under different influencing factors (Table 3), there were significant differences in the proportion of CE (*P* = 0.036), baseline NIHSS score of the recurrent stroke (*P* = 0.007), and the interval between IVT (*P* = 0.041). It indicated that a higher risk of sICH in RIVT is associated with CE, high baseline NIHSS score of the recurrent stroke, and short intervals between two IVT. There were no significant differences in gender, age, and involved arteries between the sICH group and the sICH group (*P* > 0.05).

**Table 3:** Comparison between cases with and without sICH after RIVT

Influencing factors	Non-sICH group (21 cases)	sICH group (3 cases)	*P* value
Male [cases (%)]	13 (61.90%)	1 (33.33%)	0.371
Age (year) [median (min, max)]	73.00 (42, 87)	76.00 (74, 81)	0.271
ACC involved in the index stroke [cases (%)]	14 (73.68%)	2 (66.67%)	0.636
ACC involved in the recurrent stroke [cases (%)]	17 (85.00%)	2 (66.67%)	0.453
TOAST classification			
LAA [cases (%)]	9 (47.37%)	0	0.186
CE [cases (%)]	5 (26.32%)	3 (100.00%)	**0.036**
SAO [cases (%)]	5 (26.32%)	0	0.442
Baseline NIHSS of the index stroke [median (min, max)]	9.00 (2, 19)	12.00 (6,18)	0.573
Baseline NIHSS of the recurrent stroke [median (min, max)]	10.00 (2, 24)	22.00 (22, 23)	**0.007**
OTT of the previous IVT (min) [median (min, max)]	100.00 (35, 350)	180.00 (120, 240)	0.294
OTT of the RIVT (min) [median (min, max)]	95.00 (33, 420)	150.00 (60, 240)	0.573
The interval between two IVT (day) [median (min, max)]	10.00 (2, 90)	4.00 (0.25, 6)	**0.041**
Good outcome after previous IVT [cases (%)]	20 (95.24%)	2 (66.67%)	0.239

Abbreviations: ACC = anterior cerebral circulation; LAA = large artery atherosclerotic; cardiogenic embolism; SAO = small artery occlusion; OTT = onset-to-treatment time; IVT = intravenous thrombolysis.

The proportion of CE, baseline NIHSS score of the recurrent stroke and the interval between IVT was considered the possible influencing factors for logistic regression analysis. Since the number of non-CE cases in the sICH group was 0, relevant data were excluded. In the logistic regression model (Table 4), the baseline NIHSS score of the recurrent stroke (*P* = 0.133) and the interval between IVT (*P* = 0.159) did not affect the risk of sICH.

**Table 4:** Binary logistic regression analysis of factors may affect the risk of sICH after RIVT

Influencing factors	*P* value	OR	95% CI
Baseline NIHSS score of the recurrent stroke	0.133	1.843	0.830 ~ 4.090
The interval between two IVT	0.159	0.714	0.447 ~ 1.141

Abbreviations: IVT = intravenous thrombolysis

### Risk of bias

All included studies were assessed for quality using the JBI Critical Appraisal Checklist for Analytical Cross Sectional Studies. The information is summarized in Supplementary Table S1. The overall risk of bias was assessed to be low as the included studies contain basically all recommended elements in the JBI checklist.

## Discussion

In this study, we retrospectively analyzed the cases of recurrent AIS who were retreated with RIVT within 3 months and grouped them according to whether they obtained good clinical outcomes and whether sICH occurred.

In terms of efficacy, the results showed that the differences in outcomes after the previous IVT and baseline NIHSS score of the recurrent stroke between the good and poor outcome group approached borderline statistical significance. This suggests that these two factors may have an impact on the clinical outcome after RIVT. This is also supported by a previous study by Wu et al. [6] They reviewed 61 cases of RIVT in recurrent AIS, of which 32.79% (20 cases) were within 3 months. The results showed that a low NIHSS score before RIVT was significantly associated with a good final clinical outcome. Whereas, different results were previously presented in a study by Karlinski et al. [25] Their study showed that there was no significant difference in the improvement of neurological function and 3-month prognosis between the patients with or without a history of AIS within 3 months, and there was no significant difference in NIHSS score between the two groups. This is consistent with the results of our study. Nonetheless, it should be noted that the study did not conduct further analysis of cases with a history of IVT in the previous stroke within 3 months, so the results of the study may be biased from the results of our study.

In terms of safety, several studies have explored the feasibility and safety of IVT for recurrent AIS patients with a history of stroke within 3 months, but there are still some controversies. A study by Karlinski et al. showed no significant difference in the incidence of sICH among patients receiving IVT therapy regardless of whether they had a history of stroke within 3 months [25]. However, Merkler et al. suggested that a history of stroke within the last 3 months was not associated with an increased risk of sICH in IVT, but it was associated with a significantly increased risk of death [26]. While Heldner et al. concluded that patients with a history of AIS within the last 3 months had a higher incidence of sICH (11.8% vs. 6%) and mortality (41.2% vs. 22.7%) after IVT than patients without a history of a recent stroke [27]. Ignacio et al. conducted a meta-analysis of the above three studies and found no significant differences in the incidence and mortality of sICH after IVT, regardless of whether there was a history of stroke within 3 months [28]. A considerable number of patients with a history of stroke within 3 months had IVT in the previous AIS, but there are few reports on these patients. In the study by Wu et al., sICH occurred more often in cases with thrombolytic intervals of less than 30 days than in cases with thrombolytic intervals of more than 30 days (25.0% vs. 4.9%), and high NIHSS scores before thrombolysis were significantly associated with the risk of sICH [6]. This study suggests that the interval between thrombolysis and baseline NIHSS score of the recurrent stroke should be important references for selecting patients for RIVT. Their findings were consistent with our study. Besides, Sarmiento et al. performed a retrospective analysis of 33 cases of early recurrent AIS treated with IVT, none of which had a thrombolytic interval of more than 90 days [7]. The results showed that the final clinical outcome and the incidence of sICH in patients who underwent RIVT were not significantly different from the published data [29] in patients without a recent history of AIS.

In terms of the dosage, a low dose of alteplase was used in the RIVT of two patients due to the old age (80 years) and low body weight (50 kg) of the patient in case 1 (0.9 mg/kg in the previous IVT and 0.6 mg/kg in the RIVT) and the large infarct volume of the patient in case 24 [24] (90 mg in the previous IVT and 50 mg in the RIVT), but good clinical outcomes were obtained without sICH. It has previously been suggested that a low dose (0.6 mg/kg) of alteplase, as compared with a standard dose (0.9 mg/kg), may result in a worse clinical outcome but a lower risk of sICH [30]. Since multiple factors may increase the risk of ICH, such as congestive heart failure, hypertension, an age of more than 75 years, diabetes mellitus, and low body weight, [31, 32] the conflicting relationship between the risk of bleeding and the efficacy of IVT should be balanced when choosing the dose of alteplase, especially when multiple bleeding risk factors are superimposed. However, it is debatable whether patients who undergo RIVT within 3 months can truly benefit from a lower dose of alteplase, and what is the most appropriate dosage for RIVT specifically, for which further studies are needed.

At present, aside from alteplase, tenecteplase and urokinase are also available for IVT. A recent meta-analysis which included 14 studies involving 3537 patients compared the efficacy and safety of tenecteplase with alteplase in patients with AIS [33]. The results showed no significant difference in obtaining a favorable outcome (mRS Score at 90 days ≤ 2) between tenecteplase and alteplase recipients, whereas tenecteplase recipients had a higher rate of early neurological improvement than alteplase recipients. This study indicated the use of tenecteplase as an alternative to alteplase for RIVT. Whereas, there are limited reports of tenecteplase used in RIVT within 3 months. Therefore, more studies are needed to verify its efficacy and safety. Up to now, we have not found any reports of RIVT in recurrent AIS within 3 months using urokinase as the thrombolytic agent.

There are some limitations should be mentioned to appropriately interpret the results of the present study. First, the small number of cases included in the present study inevitably leads to some bias in the results. In the aspect of efficacy, the difference between the two groups in the proportion of good clinical outcomes after the index IVT (*P* = 0.054) and in the baseline NIHSS score of the recurrent stroke (*P* = 0.056) reached borderline significance. In the aspect of safety, the baseline NIHSS score of the recurrent stroke and interval between IVT were associated with sICH in univariate analysis, but logistic regression analysis did not show their effect on the risk of sICH. Therefore, further studies with larger samples are needed to validate these conclusions. Second, if poor clinical outcomes have been shown in the previous IVT, patients may not want to receive RIVT. Some bias may occur in this situation. Third, since RIVT within 3 months for recurrent AIS is currently an off-guideline treatment, some cases may not be reported due to poor prognoses, such as severe neurological deficit symptoms or death. This may lead to some publication bias in the included studies, and the actual incidence of poor outcomes and sICH may be higher.

## Conclusions

This study suggests that RIVT may be considered in patients with recurrent AIS within 3 months when the previous IVT obtained a good outcome and the baseline NIHSS score of the recurrent stroke is low. However, a high baseline NIHSS score, a short interval between thrombolysis and cardiogenic embolism may increase the risk of sICH after RIVT. Due to the small sample size and publication bias, future studies with larger sample sizes and more rigorous designs are needed to confirm this conclusion.

### Electronic supplementary material

Below is the link to the electronic supplementary material.

Supplementary Material 1

## Data Availability

Data are available from the corresponding authors upon reasonable request.

## Abbreviations

RIVT: Repeated intravenous thrombolysis
AIS: Acute ischemic stroke
IVT: Intravenous thrombolysis
ESO: European Stroke Organization
AHA: American Heart Association
ASA: American Stroke Association
PRISMA: Preferred Reporting Items for Systematic Reviews and Meta-Analyses
CNKI: China National Knowledge Infrastructure
JBI: Joanna Briggs Institute
NIHSS: National Institutes of Health stroke scale
OTT: Onset-to-treatment time
mRS: Modified Rankin scale
sICH: Symptomatic intracranial hemorrhage
NIHSS: National Institute of Health stroke scale
NR: Not reported
LAA: Large artery atherosclerotic
CE: Cardiogenic embolism
SAO: Small artery occlusion
L: Left
R: Right
MCA: Middle cerebral artery
PCA: Posterior cerebral artery
VA: Vertebral artery
BA: Basilar artery
