# Supplementary material for: Repeated intravenous thrombolysis in recurrent ischemic stroke within 3 months: a systematic review
Source: BMC Neurol. 2023 Nov 27;23:422. doi: 10.1186/s12883-023-03472-4 (PMC10680229; doi:10.1186/s12883-023-03472-4)
Supplement: Supplementary file 1 — Supplementary Material 1 [file 12883_2023_3472_MOESM1_ESM.docx]

| **Table S1.** Quality Assessment of Included Studies. | | | | | | | | |
| --- | --- | --- | --- | --- | --- | --- | --- | --- |
| Author, year | Q1 | Q2 | Q3 | Q4 | Q5 | Q6 | Q7 | Q8 |
| Topakian et al. 2005 | Y | Y | Y | Y | Y | Y | Y | Y |
| Smadja et al. 2009 | Y | Y | Y | Y | Y | Y | Y | Y |
| Sauer et al. 2010 | Y | N | Y | Y | Y | Y | Y | Y |
| Nicoli et al. 2011 | Y | Y | Y | Y | Y | Y | Y | Y |
| Cappellari et al. 2012 | Y | Y | Y | Y | Y | Y | Y | Y |
| Brigo et al. 2012 | Y | Y | Y | Y | Y | Y | Y | Y |
| Alhazzaa et al. 2013 | Y | N | Y | Y | Y | Y | Y | Y |
| Yoo et al. 2013 | Y | N | Y | Y | Y | Y | Y | Y |
| Sposato et al. 2013 | Y | Y | Y | Y | Y | Y | Y | Y |
| Qureshi et al. 2015 | Y | N | Y | Y | Y | Y | Y | Y |
| Mechtouff et al. 2015 | Y | Y | Y | Y | Y | Y | Y | Y |
| Laible et al. 2015 | Y | N | Y | Y | Y | Y | Y | Y |
| Xiao et al. 2017 | Y | Y | Y | Y | Y | Y | Y | Y |
| Bouchal et al. 2021 | Y | Y | Y | Y | Y | Y | Y | Y |
| Černík et al. 2021 | Y | Y | Y | Y | Y | Y | Y | Y |
| Chen et al. 2023 | Y | Y | Y | Y | Y | Y | Y | Y |
| The Joanna Briggs Institute (JBI) Critical Appraisal Checklist for Analytical Cross Sectional Studies:  Q1: Were patient’s demographic characteristics clearly described?  Q2: Was the patient’s history clearly described and presented as a timeline?  Q3: Was the current clinical condition of the patient on presentation clearly described?  Q4: Were diagnostic tests or assessment methods and the results clearly described?  Q5: Was the intervention(s) or treatment procedure(s) clearly described?  Q6: Was the post-intervention clinical condition clearly described?  Q7: Were adverse events (harms) or unanticipated events identified and described?  Q8: Does the case report provide takeaway lessons?  N: No; Y: Yes; U: Unclear; N/A: Not Applicable. | | | | | | | | |
